# Maternity Homes

**Published:** 1920-02-28

**Authors:** 


THE PUBLIC WELL-BEING—*(continued)*

### Maternity Homes.

### Advice and Suggestions.

In answer to a Parliamentary question, the Minister of Health states that the Local Government Board and the Ministry of Health have for some time pressed upon local authorities, both in general circulars and in individual letters, the importance of the provision and maintenance of maternity homes, and have obtained Treasury sanction for a grant of half the approved expenditure on these purposes. A memorandum has just been issued and will be placed on sale, giving guidance as to suitable plans and equipment. During the last year or so about twenty-five such homes were established by municipal authorities, and about twenty by voluntary bodies who, as a rule, received financial assistance from local authorities. Many similar homes have been planned and are likely to be established in the near future.

This memorandum, which has been prepared for official use, comprises notes on the best designs for maternity institutions; but, while everyone is inclined to be sceptical

**THE PUBLIC WELL-BEING**—*(continued)*.

whenever the subject of building is mentioned, it is well to have a clear view of what should be aimed at and to have the whole subject seriously considered.

With regard to maternity homes, it is suggested that such should contain up to eighteen or twenty beds mainly for normal cases or cases of minor difficulty. As rest and quiet are important factors in such a home, preference should be given to a building on a reasonably secluded site with a garden attached.

Maternity hospitals having twenty-five to fifty, or an even greater number of beds, are mainly required in the large towns. They should, when possible, become the centre of the midwifery service of the district, and under suitable conditions, be available for the training of pupils and students. The site for a home or hospital of twelve to eighteen beds should not be less than an acre and a-half, while the area of a site or a hospital of twenty or thirty beds should not be less than two acres.

As to the construction of the buildings, it is stated that brickwork is probably the most economical material, while the floors should be of solid concrete construction on the ground floor and a boarded floor on the first floor, covered with good cork lino or cork carpet, which can be laid with a minimum of joining. Woodwork should be reduced to a minimum, and should be painted and varnished or finished with an enamel paint.

The period for which a woman should remain in the hospital or home should never be less than fourteen days after the birth of a child. It is desirable that the period should be extended to three weeks or even longer. Where a mother is unable to seek admission to a maternity home owing to family responsibilities some assistance should be given through "home helps," and the home or hospital may be able to recommend such women directly or give information as to where applications should be made.

It is a national duty here involved, not only to the children, but also to the mothers; for, while infant mortality has shown a progressive decline, the maternal death-rate has been more or less stationary. The two points should go side by side, and more attention should be devoted to safeguarding the lives and health of the mothers. Institutions, training, education, and provision of skilled attendants, and emphasis on the need of healthy living, are all directions to which attention must be applied more exactly than hitherto. There is an undoubted growth in the social conscience related to these matters, and the increasing share of women in our public life should still further stimulate all that goes to improvement.

There is, however, another side of this question which should not be lost sight of. The Ministry makes reference to the assistance necessary for mothers whose family responsibilities prevent them from entering a maternity home; the provision of home helps is suggested. This is good enough so far as it goes, but it does not cover that large class of women who do not care to enter maternity institutions, who would probably not wish to have home helps, and for whom the private maternity homes are too expensive. This is a class which, as in all things, has to pay for advantages provided for others, but reaps no benefits itself. Added to all its problems is the still very difficult one of getting domestic help, and in many cases even nursing help in the home. In any public plans for maternity homes provision should be made for private wards for those who can contribute something. There are some things at least in which they might be allowed some return for their money, and this seems to be one of them.

